# High-Risk Coronary Plaques and Carotid Duplex Findings in Asymptomatic Patients Undergoing Primary Prevention Assessment

**DOI:** 10.3390/jcdd13020088

**Published:** 2026-02-11

**Authors:** Lucio Addeo, Pasquale Guarini, Carlo Tedeschi, Antonio Rapacciuolo, Salvatore Severino, Mario De Michele, Milena Sidiropulos, Mattia Silvestre, Carlo Liguori, Luigi Cocchiara, Stefano Nardi, Luigi Argenziano, Vittoria Marino, Pasquale Campana, Roberto Franco Enrico Pedretti, Maurizio Bussotti, Laura Adelaide Dalla Vecchia

**Affiliations:** 1 Department of Advanced Biomedical Sciences, University of Naples Federico II, Via Sergio Pansini, 80131 Naples, Italy; addeolucio@gmail.com (L.A.); antonio.rapacciuolo@unina.it (A.R.); 2 U.O. Cardiologia, Clinica Sanatrix, 80127 Naples, Italy; guarini@iol.it (P.G.); mattiasilvestremd@gmail.com (M.S.); 3 IRCCS SYNLAB SDN, Via Emanuele Gianturco 113, 80143 Naples, Italy; carlo.tedeschi@hotmail.it; 4 Casa di Cura San Michele, Via Appia, 187, 81024 Maddaloni, Italy; dr.sseverino@gmail.com; 5 Presidio Ospedaliero San Giuseppe Moscati, Via Antonio Gramsci, 1, 81031 Aversa, Italy; demic@libero.it; 6 Ospedale Santa Maria delle Grazie, Via Domiziana, 80078 Pozzuoli, Italy; milesidi@gmail.com; 7 San Giovanni Bosco Hospital, Radiology Unit, Via Filippo Maria Briganti, 255, 80144 Napoli, Italy; carlo.liguori@gmail.com; 8 Pineta Grande Hospital, 81030 Castel Volturno, Italy; nardi.stefano@libero.it (S.N.); luigi.argenziano@pinetagrande.it (L.A.); marinovittoria723@gmail.com (V.M.); campanapasquale@gmail.com (P.C.); 9 School of Medicine and Surgery, University of Milano Bicocca, 20126 Milan, Italy; roberto.pedretti@unimib.it; 10 Cardiology Unit, Hospital of Erba, Erba (CO), 22036 Erba, Italy; 11 Department of Cardiology, IRCCS Istituti Clinici Scientifici Maugeri, 20138 Milan, Italy; maurizio.bussotti@icsmaugeri.it (M.B.); laura.dallavecchia@icsmaugeri.it (L.A.D.V.)

**Keywords:** coronary CT angiography, high-risk plaque, carotid ultrasound, calcium score, vulnerable coronary plaques, cardiovascular prevention

## Abstract

Subclinical coronary atherosclerosis is common but its biological aggressiveness and interplay with extracoronary disease in asymptomatic individuals remain unclear. We evaluated the prevalence of high-risk coronary plaques (HRPs) and their relationship with mild carotid atherosclerosis and coronary calcium in a cardiovascular (CV) high-risk cohort in primary prevention. This retrospective multicenter study enrolled 269 asymptomatic adults with multiple CV risk factors who underwent Coronary Computed Tomography Angiography (CCTA) after prior carotid duplex ultrasound (CDUS). Coronary artery disease (CAD) was graded as absent, non-obstructive (<50% stenosis) or obstructive (≥50%), and HRPs were identified by ≥1 adverse morphological feature (low attenuation, positive remodeling, napkin-ring sign, spotty calcification). Carotid disease was classified as CDUS 0 (no plaque), CDUS 1–49% (mild), or CDUS ≥ 50% (significant). Pre-specified analyses explored prevalence of HRPs across CDUS–calcium-score strata (cut-off 100 Agatston) and independent predictors within the CDUS 1–49% subgroup. CAD was absent in 31%, non-obstructive in 41%, and obstructive in 28%. HRPs were present in 30.9% of all cases, in 26.6% of non-obstructive and in 64.6% of obstructive CAD. HRPs prevalence rose step-wise from 10.0% (CDUS 0 + Ca < 100) to 27.7% (CDUS 1–49% + Ca < 100), 41.2% (CDUS 0 + Ca ≥ 100) and 59.4% (CDUS 1–49% + Ca ≥ 100). In patients with CDUS 1–49%, current smoking independently predicted HRPs (OR 2.1, 95% CI 1.0–4.5; *p* = 0.049). Nearly one-third of asymptomatic adults with high CV risk already showed HRPs. Mild carotid atherosclerosis synergized with a calcium score ≥ 100 to identify a subgroup in which six of ten individuals exhibited HRPs. Smoking was the only independent clinical correlate identified of plaque vulnerability. Combined carotid ultrasound, calcium scoring and CCTA may substantially refine primary prevention risk stratification beyond traditional factors.

## 1. Introduction

Coronary artery disease (CAD) remains the leading cause of morbidity and mortality worldwide [1]. Large-scale real-world data show that over 40% of patients suffering a first myocardial infarction had no preceding symptoms and 63% were not receiving preventive medication, underscoring the limitations of symptom-based strategies [2]. Traditionally, the diagnostic approach to CAD has been guided by the presence of symptoms and focused primarily on identifying flow-limiting stenoses using ischemia-based testing. However, mounting evidence has demonstrated that many acute coronary syndromes arise from non-obstructive lesions, particularly those with high-risk morphological features such as thin fibrous caps, large lipid cores, and positive remodeling [3]. Coronary computed tomography angiography (CCTA) has emerged as a powerful noninvasive tool capable of detecting not only the degree of coronary stenosis but also the composition and vulnerability of atherosclerotic plaques [4]. Beyond luminal stenosis, CCTA offers reproducible characterization of plaque morphology and can track therapy-induced remodeling [5]. Recent guidelines have progressively incorporated CCTA into diagnostic algorithms, especially in patients with stable chest pain and intermediate pre-test probability of CAD. Its role in asymptomatic patients, particularly in the setting of primary prevention, remains less clearly defined. A growing body of literature supports the concept that CCTA can identify subclinical atherosclerosis and stratify cardiovascular (CV) risk even in the absence of symptoms.

High-risk plaque features detected by CCTA have been associated with increased risk of future cardiac events, regardless of the severity of luminal narrowing [4]. In parallel, other non-invasive modalities such as carotid duplex ultrasound (CDUS) and coronary artery calcium scoring (CAC score) have gained traction as surrogate markers of systemic atherosclerotic burden. The coexistence of early atherosclerotic changes in multiple vascular beds may reflect a phenotype of systemic vulnerability. In hypertensive outpatients, a carotid plaque score ≥ 2 was recently associated with a four-fold higher median CAC score and more severe Coronary Artery Disease–Reporting and Data System (CAD-RADS) categories [6], supporting the concept of a systemic ‘vulnerability phenotype’ [7]. However, the interplay between subclinical carotid atherosclerosis, coronary calcification, and the presence of high-risk (i.e., vulnerable) plaques (HRPs) has not been thoroughly investigated in asymptomatic individuals undergoing primary prevention assessment. Furthermore, the identification of subclinical predictors of coronary plaque vulnerability in such patients could offer valuable insights for refining risk stratification strategies. This study aimed to evaluate the prevalence of HRPs in a high CV risk cohort of asymptomatic individuals referred for their first CCTA as part of a primary prevention strategy. We also explored how these findings may relate to carotid atherosclerosis assessed by CDUS, CAC score, and traditional CV risk factors [8].

## 2. Materials and Methods

### 2.1. Study Design and Population

We retrospectively screened all patients undergoing their first image-based coronary assessment with CCTA as part of a primary prevention strategy during the study period (1 November 2023–31 January 2025) at the participating centers (Pineta Grande Hospital, Castelvolturno (CE) and Clinica Sanatrix, Naples; Italy). Patients were consecutively included among those meeting prespecified inclusion criteria, namely the availability of a complete CCTA dataset and the performance of a CDUS examination prior to (≤6 months) the CCTA. The final study population therefore reflects consecutive eligible patients with complete imaging data. All patients were asymptomatic at the time of imaging and had no prior history of CAD and/or cerebrovascular disease, thereby representing an asymptomatic cohort, but with multiple CV risk factors. Imaging investigations had been likely recommended on the basis of clinical judgment as an individualized strategy to avoid CV events in high-risk patients (Figure S1).

Imaging investigations were not performed as part of a population screening program. Referral to CCTA was based on individualized clinical judgment in asymptomatic patients considered at high cardiovascular risk despite the absence of angina or angina-equivalent symptoms. Typical referral reasons included the coexistence of multiple cardiovascular risk factors, discordant or borderline estimated risk by traditional scores, evidence of subclinical atherosclerosis at carotid duplex ultrasound, or clinician concern for occult coronary disease not adequately captured by risk calculators alone. No uniform risk-score threshold mandated CCTA referral; however, across participating centers, imaging was consistently used as an advanced risk-stratification tool in selected high-risk primary-prevention patients.

**Figure 1:**
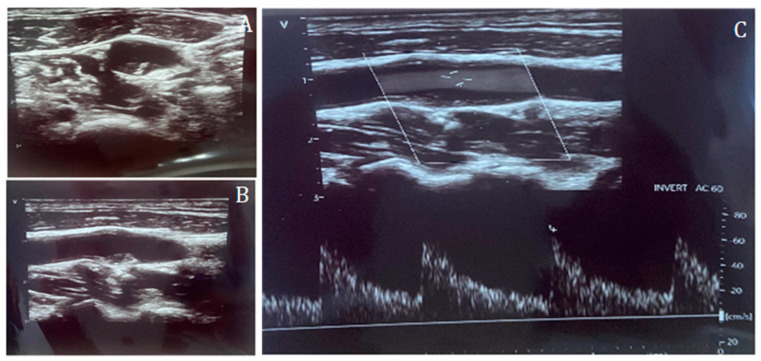
Carotid duplex ultrasound representation of a non-significant lesion of the internal carotid artery in short-axis (**A**) and long-axis (**B**), and Doppler assessment (**C**), confirming the absence of flow acceleration across the plaque.

**Figure 2:**
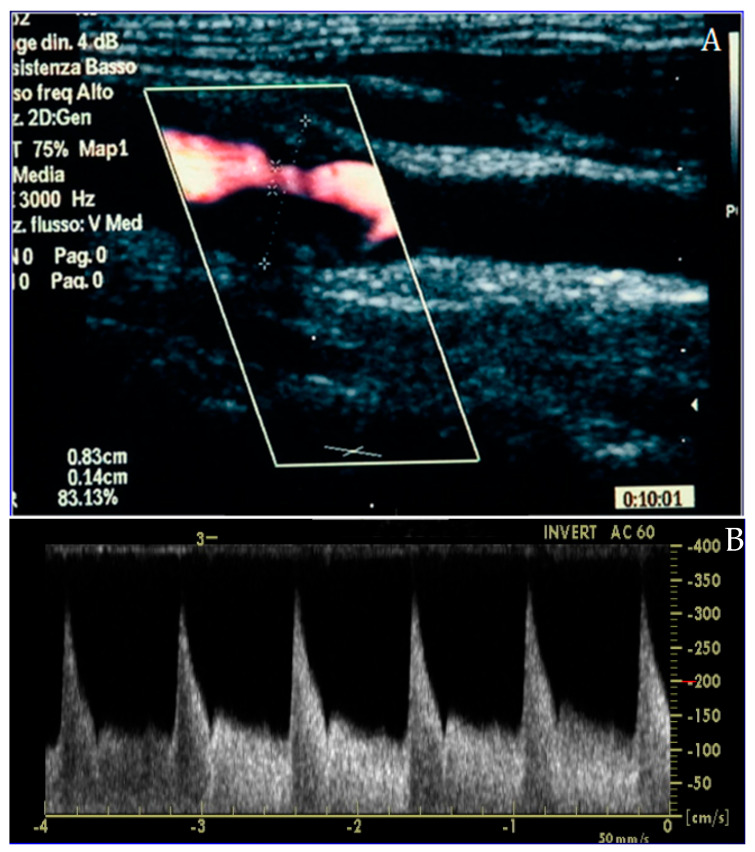
Carotid duplex ultrasound representation of a significant lesion of the internal carotid artery in long-axis (**A**) and Doppler assessment (**B**), confirming the effective flow acceleration across the plaque.

Inclusion criteria were (1) age ≥ 18 years, (2) availability of complete CCTA datasets, (3) availability of a CDUS performed prior to CCTA, and (4) availability of clinical data including age, body mass index (BMI), sex, hypertension, diabetes, smoking status, hypercholesterolemia, and family history of CV disease. Patients with history of CAD, known cardiomyopathy, angina or angina-equivalent symptoms, and chest pain were excluded. CAC score, CAD-RADS classification, and plaque morphology were recorded from CCTA reports. Carotid disease was assessed by CDUS and categorized according to the degree of stenosis: CDUS 0 (no detectable plaque), CDUS 1–49% (mild stenosis), and CDUS ≥ 50% (significant stenosis) (Figure 1 and Figure 2).

Thus, this design allowed for an evaluation of coronary and carotid atherosclerosis in an asymptomatic population with high CV risk, but no history of CAD, offering insights into the prevalence and correlates of high-risk coronary plaque features in a real-world primary prevention setting.

### 2.2. Study Endpoints and Definitions

The primary endpoints of the study were represented by (1) the presence of HRPs as detected by CCTA. These plaques presence and morphology were assessed on CCTA according to standardized qualitative criteria endorsed by the Society of Cardiovascular Computed Tomography (SCCT). Plaque was defined as any structure ≥ 1 mm^2^ within or adjacent to the coronary lumen, clearly distinguishable from the lumen and surrounding epicardial adipose tissue, and visible in at least two orthogonal planes. High-risk plaque features were identified based on established CCTA markers, including low-attenuation plaque (≤30 Hounsfield Units), positive remodeling (remodeling index ≥ 1.1), spotty calcification (<3 mm, >130 HU), and the napkin-ring sign. These qualitative criteria are supported by expert consensus documents and have been consistently associated with plaque vulnerability and adverse cardiovascular outcomes. Those plaques were consistently reported as “type H” plaques in structured imaging reports. The other primary endpoint was (2) the stratification of CAD severity based on CAD-RADS classification. Coronary arteries were classified as normal in the absence of atherosclerotic lesions (CAD-RADS 0), non-obstructive when stenosis was <50% (CAD-RADS 1–2), and obstructive when stenosis was ≥50% (CAD-RADS ≥ 3). The study also explored the relationship between coronary plaque vulnerability and markers of systemic atherosclerosis by examining the prevalence of HRPs across combinations of CDUS findings and CAC score. In particular, carotid atherosclerosis was categorized as no disease (CDUS 0) and mild stenosis (CDUS 1–49%), while the CAC burden was stratified using a cutoff of 100 Agatston units. Among patients with CDUS 1–49%, a dedicated multivariate analysis was conducted to identify independent clinical predictors of HRPs. Lastly, although not powered for inferential analysis, the subgroup of patients with significant carotid stenosis (CDUS ≥ 50%) was described separately. This descriptive endpoint focused on reporting the frequency of elevated CAC score and the presence of HRPs within this subgroup, to provide additional clinical insight into the coexistence of advanced carotid and coronary atherosclerosis.

### 2.3. Imaging Description

**Figure 3:**
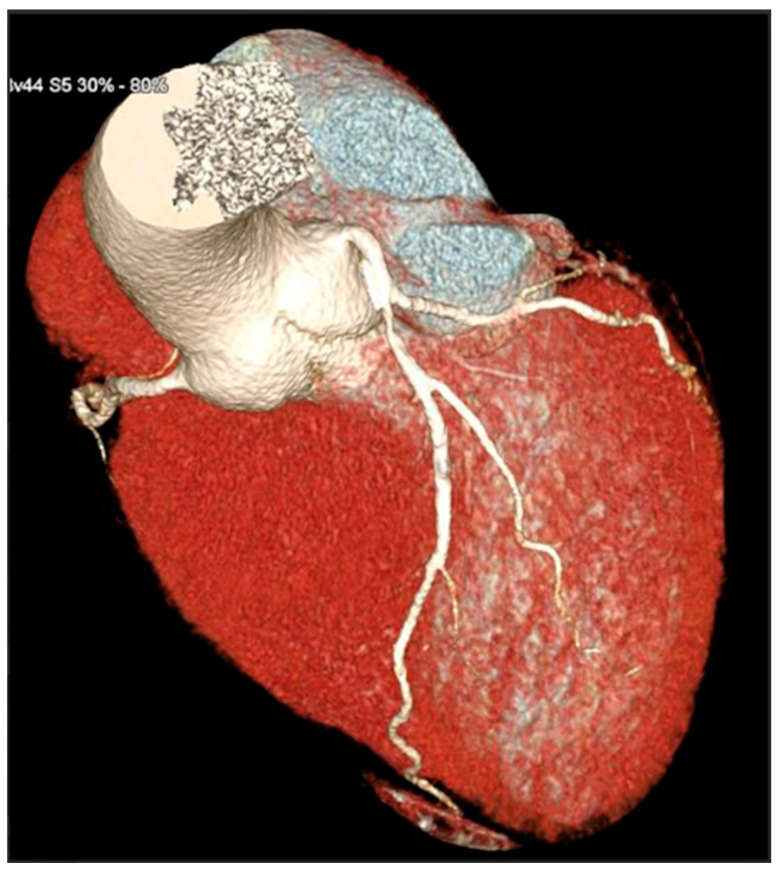
3D reconstruction of coronary CT angiography showing a significant stenosis in the proximal segment of the left anterior descending coronary artery.

CCTA scans were acquired using a 128-slice single-tube Siemens CT scanner (Definition AS+, Siemens Healthcare, Forchheim, Germany) in a retrospectively ECG-gated sequential mode. Acquisition parameters included collimation of 128 × 0.6 mm, gantry rotation time of 0.3 s, and application of automatic tube current modulation (CARE Dose 4D) with voltage settings adapted to body habitus (typically 100–120 kV). The scanning range extended from the carina to the diaphragm, with ECG triggering centered at 70% of the R-R interval. Patient preparation included oral administration of a beta-blocker (e.g., atenolol) one day before the scan to target a heart rate < 65 bpm. On the day of the scan, sublingual nitroglycerin (0.4–0.8 mg) was administered 5 min prior to acquisition to achieve coronary vasodilation. Contrast injection consisted of 70–75 mL of Iopamidol (370 mgI/mL) followed by 40 mL of saline flush, injected at 5.5 mL/s via an antecubital vein. Bolus tracking was performed at the level of the ascending aorta, with a threshold of 150 Hounsfield Units to initiate the scan. Image reconstruction employed a slice thickness of 0.6 mm with 0.4 mm increment and iterative reconstruction algorithms (SAFIRE or ADMIRE), using a dedicated coronary kernel (e.g., B26f or B40f BV 44f). If necessary, motion correction was performed using multi-segment reconstruction. Post-processing included multiplanar reconstructions (MPRs), curved planar reconstructions (CPRs), maximum intensity projections (MIPs), and 3D volume rendering (VR), conducted on Syngo.via workstations. Coronary artery analysis followed the American Heart Association 16-segment model. Coronary stenosis was classified as significant when luminal narrowing exceeded 50% or 70%, based on consensus by at least two out of three experienced CCTA readers. CCTA examinations were interpreted by experienced cardiovascular imaging readers with >5 years of dedicated CCTA experience and annual volumes exceeding 300 studies per reader. Coronary stenosis severity and plaque morphology were assessed by consensus of at least two readers; in case of disagreement, a third senior reader adjudicated. Readers had access to standard clinical referral information but were blinded to carotid duplex ultrasound findings and CAC score at the time of plaque morphology assessment. A 3D reconstruction allowed for a comprehensive reconstruction of the anatomy of coronary arteries (Figure 3).

**Figure 4:**
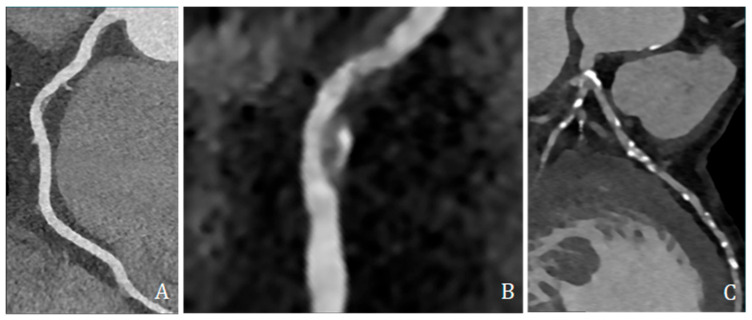
Coronary CT angiography showing different plaques and morphologies along different coronary arteries. (**A**,**B**) show predominantly non-calcified plaque, with low density, positive remodeling, and spotty calcification; (**C**) shows several predominantly calcified plaques all along coronary arteries, mainly the left anterior descending artery.

High-risk plaque features were assessed and included low-attenuation plaque (LAP, ≤30 HU), spotty calcification (SC, dense foci > 130 HU within non-calcified tissue and <3 mm), and positive remodeling (PR, remodeling index ≥ 1.1) (Figure 4).

Radiation dose was calculated based on the dose-length product (DLP) multiplied by a standard conversion coefficient of 0.017 mSv/(mGy.cm), as per Fleischner Society recommendations [9]. This protocol ensured a balance between diagnostic image quality and radiation exposure. CDUS was performed using a high-resolution ultrasound system with a 9 MHz linear-array transducer. Participants were examined supine, with slight neck extension and contralateral rotation. The common carotid artery, carotid bulb, and internal and external carotid arteries were assessed bilaterally in longitudinal and transverse planes. B-mode imaging was used to measure intima–media thickness and identify plaques; color Doppler evaluated luminal patency and flow; and pulsed-wave Doppler recorded peak systolic and end-diastolic velocities with an insonation angle ≤ 60° and the sample volume centered in the flow stream. Plaque was defined as a focal structure encroaching into the lumen ≥ 0.5 mm or ≥50% of the surrounding intima–media thickness, or with maximal thickness > 1.5 mm. Internal carotid artery stenosis was categorized as 0%, 1–49%, or ≥50% based on standard duplex velocity criteria.

### 2.4. Statistical Analysis

Statistical analyses were performed using Python (version 3.10) with the Pandas, SciPy, and StatsModels libraries. Continuous variables were reported as mean ± standard deviation and compared using Student’s *t*-test or Mann–Whitney U test, as appropriate, based on normality assumptions. Categorical variables were presented as absolute counts and percentages and compared using the chi-square test or Fisher’s exact test where indicated. The prevalence of HRPs, CAD severity (absent, non-obstructive, and obstructive), and CAC score categories was calculated for the entire cohort and across subgroups defined by CDUS findings and clinical variables. The association between carotid disease and the presence of HRPs was explored by stratifying patients according to CDUS category (CDUS 0, CDUS 1–49%, and CDUS ≥ 50%) and CAC score (<100 vs. ≥100). Given the retrospective and exploratory nature of the study, no formal a priori sample size or power calculation was performed. The analyses were intended to describe prevalence patterns and generate hypotheses rather than to test predefined causal associations. Among patients with CDUS 1–49%, a binary logistic regression model was then used to identify independent predictors of plaque vulnerability in this subgroup. Odds ratios (ORs) with corresponding 95% confidence intervals (CIs) and *p*-values were reported. A two-sided *p*-value < 0.05 was considered statistically significant. A separate descriptive analysis was conducted on the subgroup of patients with significant carotid stenosis (CDUS ≥ 50%) to report the frequency of elevated CAC scores and HRPs. Due to the limited number of observations in this group, no inferential statistics were applied.

### 2.5. Ethics Approval

Ethical review and approval were waived for this study because of its retrospective design and the complete anonymization of patients’ data. Informed consent was not required in accordance with institutional policies and national regulations, as no identifiable personal information was used and no interventions beyond standard clinical care were performed.

## 3. Results

A total of 269 asymptomatic patients undergoing CCTA for primary prevention were included in the study. Women accounted for 99 patients (36.8%) of the study population. Baseline characteristics are shown in Table 1.

Overall, 51.0% of patients were receiving at least one lipid-lowering medication. Specifically, statin therapy was recorded in 97/269 (36.1%), ezetimibe in 99/269 (36.8%), PCSK9 inhibitors in 3/269 (1.1%), and bempedoic acid in 2/269 (0.7%). Concomitant statin plus ezetimibe therapy was present in 61/269 (22.7%). An antithrombotic therapy was present in 8/269 (3.0%) (Table 2).

**Figure 5:**
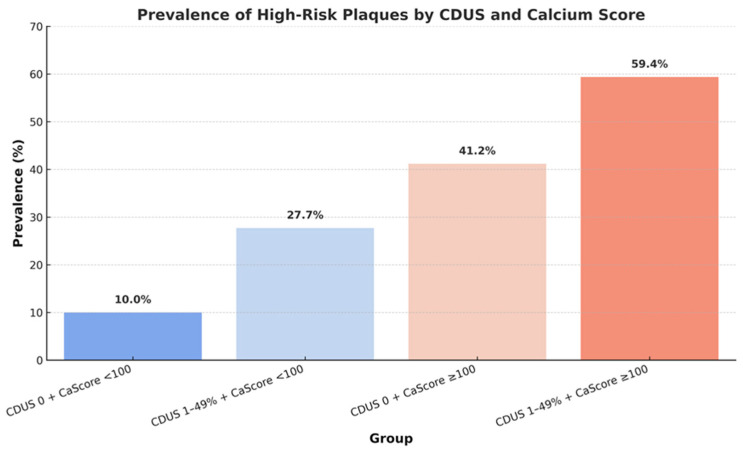
Prevalence of high-risk coronary plaques across strata defined by carotid duplex ultrasound (CDUS) and coronary calcium (Ca) score.

CAD was classified as absent in 31.3% of cases, non-obstructive (<50% stenosis with CAD RADS 1–2) in 40.7%, and obstructive (≥50% stenosis with CAD RADS ≥ 3) in 28% of patients. The overall prevalence of HRPs was 30.9%. Among patients with non-obstructive disease, 26.6% exhibited at least one vulnerable plaque, while in patients with obstructive disease, 64.6% showed at least one vulnerable plaque. The prevalence of HRPs increased progressively according to CDUS and CAC score grading: 10.0% in patients with CDUS 0 and CAC score < 100, 27.7% in CDUS 1–49% with CAC score < 100, 41.2% in CDUS 0 with CAC score ≥ 100, and 59.4% in CDUS 1–49% with CAC score ≥ 100 (Figure 5).

**Figure 6:**
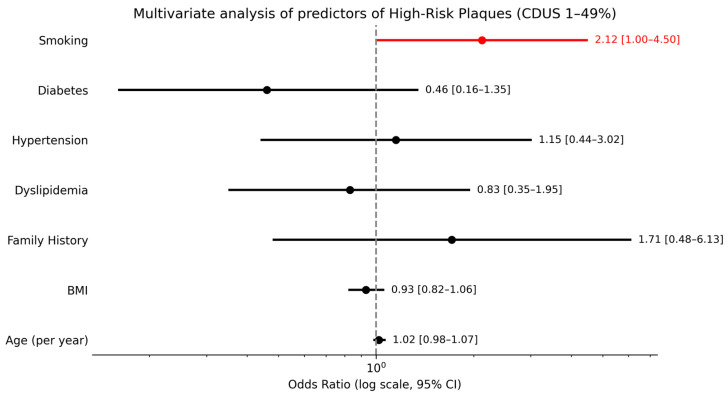
Multivariate analysis of predictors of high-risk plaques in patients with CDUS 1–49%.

A separate descriptive analysis of the six patients with significant carotid stenosis (CDUS ≥ 50%) revealed that all had elevated CAC scores, and 4 out of 6 (66.7%) presented at least one vulnerable plaque. In the subgroup of patients with CDUS 1–49%, smoking was the only independent predictor of HRPs (OR ≈ 2.12; *p* = 0.049), while age (per year), hypertension, BMI, diabetes, hypercholesterolemia, and family history showed no significant associations (Figure 6).

## 4. Discussion

The present study shows three key findings which, taken together, may help to clarify the systemic nature of subclinical atherosclerosis and its practical implications for primary prevention. Firstly, almost one in three adults undergoing CCTA for the first time for primary prevention already displayed at least one vulnerable plaque. This prevalence is higher than that reported in cohorts undergoing general screening [10,11], but lower than that observed in cases of acute chest pain, placing it halfway between the two spectra described in previous coronary CT investigations [12]. A similar intermediate prevalence (21%) has been reported among healthy commercial aircrew screened with CCTA, further positioning our cohort between population studies and symptomatic settings [13]. It is important to emphasize that in this retrospective analysis of data, CCTA was not used as a screening tool for the general population, nor in the setting of established chronic coronary syndrome, but as an advanced risk stratification tool in asymptomatic individuals considered to be at high CV risk. In line with earlier data on populations with stable chest pain, we also confirmed that vulnerable plaque burden is common even when luminal stenosis is non-obstructive, reinforcing the concept that it is the plaque phenotype, rather than diameter narrowing alone, that drives future events [12]. A large longitudinal analysis of stable symptomatic patients also demonstrated that low attenuation plaque volume is more effective than traditional stenosis metrics in predicting myocardial infarction over five years [14], lending external validity to the clinical relevance of the high burden of vulnerable plaque we observed in asymptomatic individuals. Secondly, we found a stepwise increase in high-risk-plaque prevalence, from 10% to almost 60%, when mild carotid atherosclerosis and a CAC score ≥ 100 co-existed. This gradient indicates a synergistic interplay between systemic plaque burden and coronary calcification. This is in line with current evidence [15] and mirrors recent findings in hypertensive cohorts where an ultrasound-derived carotid plaque score independently tracked both calcium burden and CAD-RADS severity on CCTA [7]. Our data extend these observations by showing that even low-grade (1–49%) carotid disease, when combined with an elevated calcium score, identifies a subgroup with a high prevalence of morphologically HRPs despite the absence of symptoms or obstructive stenoses, thus reinforcing carotid evaluation as a powerful tool for better stratifying and predicting patients at higher risk of CV events [16]. Thirdly, smoking was the only independent clinical predictor of plaque vulnerability in patients with mild carotid disease, highlighting a strong association between active smoking and plaque vulnerability. With regard to the role of obesity, our analysis did not identify it as an independently related factor, which is partly consistent with current evidence [17], but which should nevertheless be further investigated. These findings reinforce guideline recommendations that place smoking cessation at the forefront of CV risk reduction strategies [18]. Given the particularly high prevalence of HRPs among smokers in our cohort, aggressive counseling and early initiation of targeted preventive therapies appear warranted in this subgroup. Importantly, patients of this study often presented with a combination of multiple risk factors rather than a single dominant determinant, leading clinicians to adopt a personalized and advanced diagnostic approach even in primary prevention, based on a judgment of at high CV risk despite the absence of symptoms. In line with our observation that mild carotid atherosclerosis and elevated CAC identify individuals with a high prevalence of morphologically HRPs, the 2025 ESC/EAS Focused Update formally endorses subclinical atherosclerosis detected by imaging and increased CAC as risk modifiers to refine CV risk stratification in individuals at moderate risk or near treatment decision thresholds [19]. Taken together, these findings support the complementary use of carotid ultrasound, CAC score and CCTA to refine primary-prevention risk stratification. Combining a rapid carotid scan with calcium score measurement may help identify asymptomatic individuals who already carry dangerous coronary plaque features and who could therefore benefit most from intensive lifestyle and pharmacological interventions. Emerging pipelines based on photon-counting CT and radiomics artificial intelligence promise to overcome current resolution constraints and provide individualized risk prediction [13].

Some limitations of the study should be considered. Its retrospective design inherently limits causal inference and may introduce selection bias, as inclusion was dependent on the availability of complete CCTA datasets and a prior CDUS examination. The study population represents consecutive eligible patients rather than an unselected consecutive referral cohort. Secondly, although all patients were asymptomatic for angina or cerebrovascular events at the time of imaging and had no prior diagnosis of CAD, the cohort reflects a clinically selected, high-risk population referred for refined CV risk assessment. Therefore, the findings should not be extrapolated to the asymptomatic general population or interpreted as supporting population-based screening strategies. Furthermore, detailed information on the intensity, duration, and effectiveness of CV risk factor control, including longitudinal levels of cholesterol or other therapeutic targets, was not consistently available due to the retrospective nature of data collection across multiple referral centers. This limitation may have attenuated the observed associations between traditional risk factors and plaque vulnerability and should be taken into account when interpreting multivariate analyses. This study was exploratory and was not designed to formally test hypotheses; thus, inferential analyses should be considered as hypothesis-generating. In addition, the small number of patients with significant carotid stenosis limited the ability to perform robust statistical analyses in this subgroup. Finally, although imaging acquisition and reporting were standardized in two tertiary cardiovascular imaging centers, patients were referred from multiple institutions, which, however, may reflect real-world heterogeneity in referral practices.

## 5. Conclusions

Despite the limitations, these findings may contribute to improving primary CV prevention in daily practice. In fact, a substantial proportion of asymptomatic adults with multiple risk factors for CAD undergoing CCTA for the first time already had HRPs, even without obstructive disease. Vulnerability increased significantly when mild carotid atherosclerosis was associated with a high CAC score, highlighting the systemic nature of early atherosclerosis. Smoking was found to be the only clinical trait that independently predicted plaque instability. These findings should be interpreted as signals for enhanced risk stratification rather than evidence supporting screening strategies. Prospective studies incorporating longitudinal outcomes and therapy response are required for validation. In this specific setting, the combination of CDUS, CAC score, and CCTA may help unmask a subgroup with a high prevalence of morphologically HRPs.

## Figures and Tables

**Table 1:** Baseline characteristics of the study population.

Variable	Patients (269)
Age, years (*mean* ± *SD*)	64.2 ± 10.5
Females, *n* (%)	99 (36.8)
BMI, (*mean* ± *SD*)	27.34 ± 3.08
Current Smoking, *n* (%)	107 (39.8)
Hypertension, *n* (%)	183 (68)
Diabetes, *n* (%)	41 (15.2)
Dyslipidemia, *n* (%)	153 (56.9)
Family History, *n* (%)	38 (14.1)
Calcium score, (*mean* ± *SD*)	259.2 ± 476.4
HRPs, *n* (%)	83 (30.9)
CAD-RADS 0, *n* (%)	84 (31.3)
CAD-RADS 1–2, *n* (%)	109 (40.7)
CAD-RADS ≥ 3, *n* (%)	75 (28.0)
CDUS 0, *n* (%)	151 (56)
CDUS 1–49%, *n* (%)	111 (41.3)
CDUS 50–100%, *n* (%)	6 (2.2)

Abbreviations, BMI: body mass index; HRPs: high-risk coronary plaques; CAD-RADS: Coronary Artery Disease–Reporting and Data System; CDUS: Carotid duplex ultrasound.

**Table 2:** Baseline preventive pharmacological therapy in the study population (N = 269).

Preventive Therapy	*n* (%)
Any lipid-lowering therapy	137 (51.0)
Statin therapy	97 (36.1)
Ezetimibe	99 (36.8)
Statin + ezetimibe combination	61 (22.7)
PCSK9 inhibitor	3 (1.1)
Bempedoic acid	2 (0.7)
Antithrombotic therapy	8 (3.0)

## Data Availability

The data presented in this study are available on request from the corresponding author.

## Supplementary Materials

The following supporting information can be downloaded at https://www.mdpi.com/article/10.3390/jcdd13020088/s1, Figure S1: Study Flow Diagram.

## Abbreviations

The following abbreviations are used in this manuscript:

BMI	Body Mass Index
CAD	Coronary Artery Disease
CAD-RADS	Coronary Artery Disease–Reporting and Data System
CAC	Coronary Artery Calcium
CAC score	Coronary Artery Calcium score
CDUS	Carotid Duplex Ultrasound
CCTA	Coronary Computed Tomography Angiography
HRPs	High-Risk Plaques/vulnerable coronary plaques
