# Supplementary material for: High-Risk Coronary Plaques and Carotid Duplex Findings in Asymptomatic Patients Undergoing Primary Prevention Assessment
Source: J Cardiovasc Dev Dis. 2026 Feb 11;13(2):88. doi: 10.3390/jcdd13020088 (PMC12941285; doi:10.3390/jcdd13020088)
Supplement: Supplementary file 1 [file jcdd-13-00088-s001.zip › jcdd-4127850-supplementary.pdf]

Figure S1. Study Flow Diagram

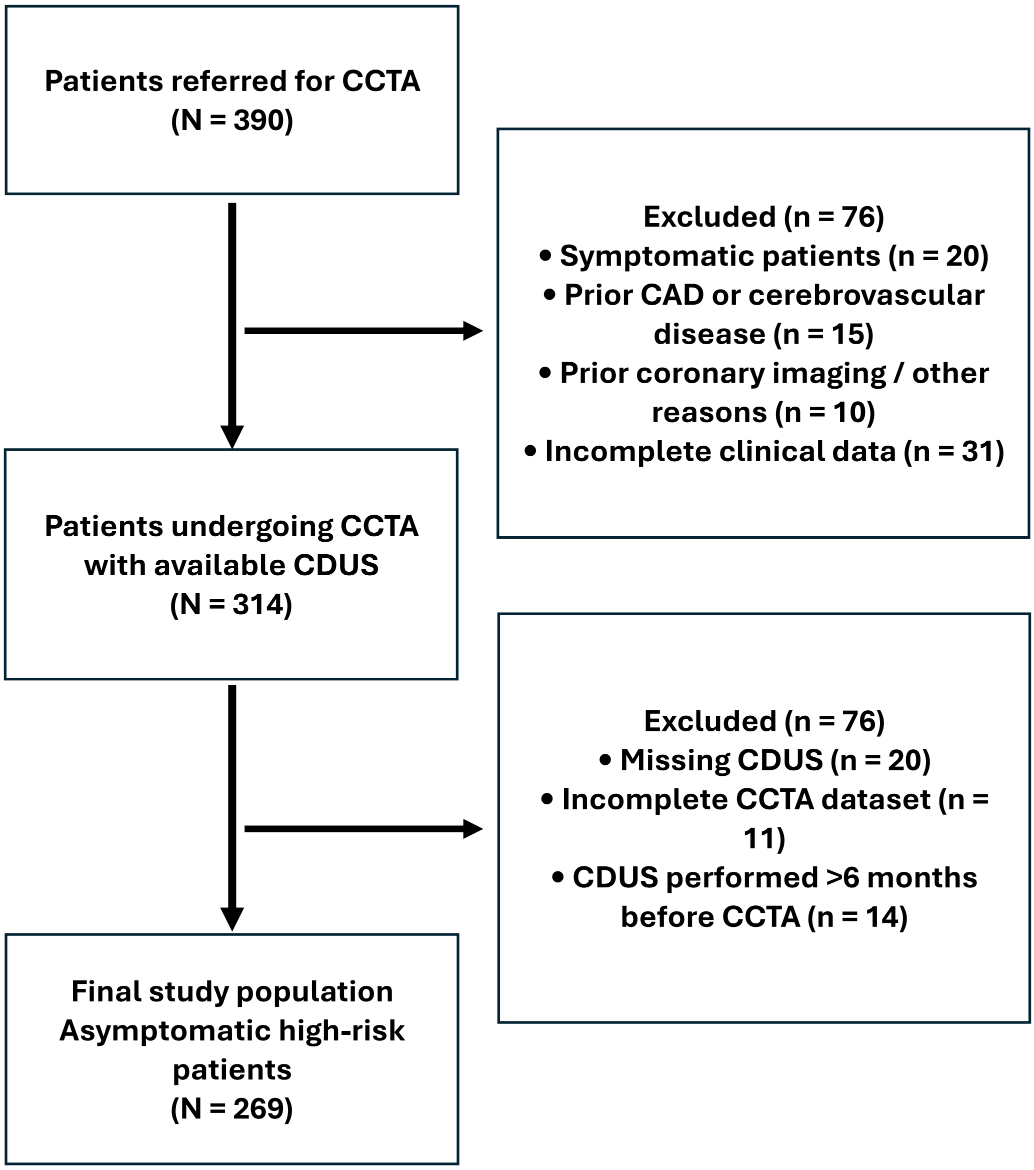

Figure S1. Study flow diagram illustrating patient selection. Patients referred for coronary computed tomography angiography (CCTA) during the study period were screened. After exclusion of symptomatic individuals, those with prior coronary or cerebrovascular disease, prior coronary imaging, or incomplete imaging data, a final cohort of 269 asymptomatic high-risk patients with available carotid duplex ultrasound (CDUS) and complete CCTA datasets was included.
